# PET-CT and RNA sequencing reveal novel targets for acupuncture-induced lowering of blood pressure in spontaneously hypertensive rats

**DOI:** 10.1038/s41598-021-90467-1

**Published:** 2021-05-26

**Authors:** Jing Li, Chong Peng, Dongjian Lai, Yajing Fang, Daihong Luo, Zunming Zhou, Chenyun Li, Xinsheng Lai

**Affiliations:** 1 Integrative Cancer Centre, The First Affiliated Hospital of Guangzhou University of Chinese Medicine, Guangzhou, 510405 Guangdong China; 2 Postdoctoral Research Station of Guangzhou University of Chinese Medicine, Guangzhou, 510405 Guangdong China; 3 Rehabilitation Center, The First Affiliated Hospital of Guangzhou University of Chinese Medicine, Guangzhou, 510405 Guangdong China; 4 Clinical School of Acupuncture and Rehabilitation, Guangzhou University of Chinese Medicine, Guangzhou, 510405 Guangdong China; 5 Department of Gastroenterology, The First Affiliated Hospital of Guangzhou University of Chinese Medicine, Guangzhou, 510405 Guangdong China; 6 Shenzhen Hospital of Beijing University of Chinese Medicine, Shenzhen, 518100 Guangdong China

**Keywords:** Genomic engineering, Molecular biology, Systems biology

## Abstract

Manual acupuncture (MA) can be used to manage high blood pressure; however, the underlying molecular mechanism remains unknown. To explore the mechanism of acupuncture in the treatment of hypertension, Wistar Kyoto rats (WKYs) and spontaneously hypertensive rats (SHRs) were subjected to either MA stimulation or the corresponding sham procedure as a negative control (Sham-MA) for 1 week. PET-CT scans, transcriptomics and molecular biology were used to evaluate the effect of MA. The results show that MA can regulate blood pressure in SHRs, change the glucose metabolism of the paraventricular hypothalamus (PVH), and affect the mRNA and protein expression levels of differentially expressed genes in the PVH. These genes may lower blood pressure by regulating angiotensin, endothelial function and inflammation. These findings reveal that MA regulates multiple biological processes and genes/proteins of the PVH, and provide a solid theoretical basis for exploring the mechanisms by which MA regulates hypertension.

## Introduction

In most international guidelines, the measurement for diagnosing hypertension is a systolic blood pressure no less than 140 mmHg and/or diastolic blood pressure no less than 90 mmHg^1^. Recently, a new consensus on the diagnosis of hypertension was formed in the 2017 ACC/AHA Hypertension Guideline and from the 2018 ESC/ESH Guideline^2,3^. Guidelines suggest that blood pressure greater than 140/90 mmHg is not the only cut-off value for diagnosing hypertension, in special populations, such as patients suffering from CVD, CKD, diabetes and other diseases. In the context of these conditions, hypertension can be diagnosed when blood pressure is greater than 130/80 mmHg. To date, the reasons for the development of hypertension have mainly been related to increased exposure to environmental factors that cause high blood pressure, including excessive consumption of salt, alcohol, and calories. Elevated blood pressure remains the largest single factor in the global burden of disease and mortality, contributing to 94 million deaths each year according to the latest data^4^. The global prevalence of high blood pressure is expected to increase by approximately 10% in the next two decades, corresponding to an estimate of 560 million people who will suffer from hypertension^5^. If hypertension is not diagnosed and managed in time, it can lead to myocardial infarction, stroke, kidney failure, and even death^6–8^.

Acupuncture is an effective alternative therapy for the treatment of hypertension^9–11^. Our previous animal trials showed that manual acupuncture (MA) at the KI3 position can effectively reduce systolic blood pressure (SBP) and diastolic blood pressure (DBP) in spontaneously hypertensive rats (SHRs)^12^. Moreover, our previous PET-CT study revealed that acupuncture may regulate blood pressure by changing the brain glucose metabolism of SHRs^13^. However, the underlying molecular mechanisms of acupuncture treatment for hypertension have not been widely described.

RNA sequencing is a new technical method that locates and quantifies the transcriptome through microarrays, which can digitally measure the presence and quantity of transcripts^14,15^. Recent transcriptomic studies have found that acupuncture affects the mRNA levels of multiple differentially expressed genes (DEGs) in the rat brain and regulates multiple biological processes, including inflammation, oxidative stress, and vascular endothelial function^16,17^. However, it has not been reported which signalling pathways and genes in specific brain regions are affected by MA at KI3.

Here, we studied the changes in cerebral glucose metabolism of 15-week-old SHRs after MA at KI3 and performed transcriptomic sequencing after isolating the paraventricular hypothalamus (PVH), which is the target brain region of acupuncture. First, we determined the DEGs, then we annotated the identified genes according to their Gene Ontology (GO) and Kyoto Encyclopedia of Genes and Genomes (KEGG) pathway classification, followed by verifying the expression levels of these DEGs by qPCR and western blot. This is a promising transcriptome study of the PVH after acupuncture at KI3 and may help us understand the role of acupuncture in the treatment of hypertension.

## Materials and methods

### Ethical statement

Experimental protocols and procedures were approved by the Experimental Animal Ethics Committee of Guangzhou University of Chinese Medicine (ref. S2017003). All experiments were performed in accordance with the ARRIVE guidelines (http://www.nc3rs.org.uk/page.asp?id=1357). All methods were performed in accordance with the relevant guidelines and regulations.

### Experimental animals

Male SHRs and WKY rats (15 weeks old, 300 ± 20 g), were purchased from Beijing Vital River Laboratory Animal Technology Co., Ltd (Beijing, China), had free access to food and water at room temperature 20–22 ℃ with a 12 h light/dark cycle.

### Groups and acupuncture treatment

WKY rats were used as the controls (WKY, n = 10). SHRs were randomly assigned to three groups: SHRs (SHRs, n = 10) without treatment, SHRs with manual acupuncture (MA) treatment at acupoint KI3 (MA, n = 10), and SHRs receiving Sham-MA treatment (Sham-MA, n = 10). The position of the KI3 acupoint was selected as the space between the tip of the medial malleolus and the attachment of the Achilles tendon. The position of the sham acupoint was selected as the space between the 3rd and 4th toes on the back of the foot. In short, an acupuncture needle (13 mm × 0.25 mm, Suzhou Hualun Medical Equipment Co., Ltd., China) was inserted into the KI3 or sham acupoint to a depth of 6 ± 1 mm, and rotated at a frequency of 90 ± 5 rotations/min and an angle of 120 ± 5°. The MA and Sham-MA groups received acupuncture for 7 consecutive days for 10 min (acupuncture on both sides, 5 min per side) per day.

### Blood pressure measurement

Systolic blood pressure (SBP) and diastolic blood pressure (DBP) were measured with a blood pressure monitor (CODA7m, Kent Scientific Corporation, Torrington, Connecticut, USA) according to the method described previously^18^ at 30, 60, and 90 min after the first day of therapy or 30 min after each day’s acupuncture treatment in the 7-day therapy. To train and improve the adaptability of the animals, rats were placed in the holder for 15 min every day for 3 days before the study. In addition, rats were preheated for 5 to 10 min until the tail temperature reached 34–36 °C before the blood pressure was measured.

### PET-CT scanning

All PET-CT scans were performed on the animal molecular imaging research platform of Sun Yat-sen Medical College. All rats were injected with 1.5 mci/kg ^18^F-FDG into the tail vein once after acupuncture treatment on day 7. When the injection was completed, rats were placed on a lead brick. Five minutes before the start of the PET-CT scan, rats were transferred to a PET-CT scanner, anaesthetized in 5% isoflurane and 100% oxygen and subjected to PET-CT scans 30 min after acupuncture. After the FDG-PET image was acquired, it was reconstructed through a 128 × 128 × 159 matrix and filtered projection.

After the PET-CT scan, all rats were anaesthetized and sacrificed by intraperitoneal injection of 30 mg/kg sodium pentobarbital.

### Tissue processing

At 24 h after the 7-day treatment, rats were euthanized and transcardially perfused with 50 ml of 0.1 M phosphate buffered saline (PBS). The PVHs were quickly isolated on ice, frozen in liquid nitrogen, and then stored at − 80 °C. The target coordinates for PVN were from Bregma − 2.80 mm to Bregma − 2.30 mm, − 0.3 mm lateral to the midline, 0 mm to 2.0 mm from ventral to caudal, and 8.0 mm to 10.0 mm from caudal to ventral according to The Rat Brain Atlas of Paxinos and Watson^19^. The PVHs that were used for RNA-Seq analysis were the WKY, SHR, MA and Sham-MA groups. Total RNA was extracted from the PVH by using the RNeasy Mini Kit (74104, Qiagen, Beijing, China) according to the manufacturer’s protocol, and an Agilent 2100 Bioanalyzer (Agilent RNA 6000 Nano Kit) was used to perform quality control for total RNA.

### RNA-Seq analysis

Transcriptome sequencing was performed by BGI Co., Ltd., Shenzhen, China (http://www.genomics.cn/). Briefly, DNA libraries were constructed using the TruSeq stranded mRNA library preparation kit (Illumina, San Diego, CA, USA) according to the manufacturer’s instructions. Paired-end reads of 100 bp were read, and the DNA libraries were sequenced on the BGISEQ-500 platform for sequence data analysis.

### Quality control for raw data

The raw data for sequencing included low-quality, adaptor-polluted material with a high content of unknown base (N) reads. These reads were removed before data analysis to ensure the reliability of the results. The Q20, Q30, and clean read ratios were calculated, and subsequent analyses were based on these clean reads.

### Differential expression analysis

Clean reads were mapped to the reference using Bowtie2^20^, and the gene expression level was calculated with RSEM^21^. DEGs with DESeq2 were detected as requested^22^. DEGs were chosen according to the parameters with a fold change ≥ 2 and an adjusted p-value (p ≤ 0.05). Then, the Pearson correlation between all samples was calculated using cor, and hierarchical clustering between all samples was performed using hclust. Gene expression was compared between SHRs and WKYs, between the MA and SHR groups, and between the Sham-MA and SHR groups. Volcano plots, scatterplots and heatmaps were generated using R version 3.6.0 (https://cran.r-project.org/bin/windows/base/old/3.6.0/). The overlap in upregulated and downregulated gene expression between the different groups was analysed using Venn online software VENNY version 2.1 (https://bioinfogp.cnb.csic.es/tools/venny/index.html). GO and KEGG classification and functional enrichment were performed for all identified DEGs. GO and KEGG analyses were performed using enrichment analysis with the phyper function in R version 3.6.0 (https://cran.r-project.org/bin/windows/base/old/3.6.0/).

### Real-time PCR

Eight DEGs were selected for validation using qRT-PCR. cDNA was produced from hypothalamic mRNA (2 μg) using an Invitrogen SuperScript II Reverse Transcriptase reagent kit (Takara, Shanghai, China). OneStep RT-PCR Enzyme mix (Qiagen, Beijing, China) was used for qRT-PCR on an ABI ViiA 7 PCR System (Thermo Fisher Scientific, USA). The Ct value in the reaction was collected using a corrected threshold setting. β-actin was used as the internal reference gene to confirm gene expression levels, and the relative quantification was determined using the 2^−ΔΔCt^ method. The primers used for qRT-PCR validation are shown in Table 1.

**Table 1:** Primer sequence information.

Gene	Primer sequences
*Ednra*	F:GCTCTAGATAGGTAGCAACGTGGCTT
*Ednra*	R:GCTCTAGAGCCCCAAAACTTGTCAAC
*Ccr5*	F:TCCTGACCACCTTCCAGGAA
*Ccr5*	R:GCAGCAGTGTGTCATCCCAA
*Angptl2*	F:TGTCAACTCCAAAGAGCCCG
*Angptl2*	R:GTCTCGATCTGCCGCTTCTG
*Gnb3*	F:TCTACAACCTCAAATCCCGC
*Gnb3*	R:TCTCAATGTCCCACAAGGC
*Erbb2*	F:GAGACAGAGCTAAGGAAGCTGA
*Erbb2*	R:ACGGGGATTTTCACGTTCTCC
*Klotho*	F:TCCCTCCTTTACCTGAGAAC
*Klotho*	R:CGGATGGCAGAGAAATCAAC
*Gpr81*	F:GGCTGAGAAAAGCGGTATGA
*Gpr81*	R:TCGTTAACTCTCTCCGAGCTAGA
*Cyp1b1*	F:CACTGCCAACACCTCTGTCTT
*Cyp1b1*	R:CAAGGAGCTCCATGGACTCT
*β-actin*	F:TCACCCACACTGTGCCCATC
*β-actin*	R:AGCTGTAGCCACGCTCGGTC

### Western blot

Western blot was performed for PVH protein detection. Fifty micrograms of PVH protein was separated by SDS and blotted onto PVDF membranes. After the membrane was blocked with 5% skim milk at room temperature for 2.5 h, it was incubated overnight with anti-Angptl2 (1:1000, Cat No. 12316–1-AP), anti-Erbb2 (1:1000, Cat No. 18299–1-AP), anti-Klotho (1:1000, Cat No.28100–1-AP), anti-Ednra (1:1000, Cat No. Ag24788), anti-Ccr5 (1:1000, Cat No. 17476–1-AP), anti-Gnb3 (1:1000, Cat No. 10081–1-AP), anti-Gpr81 (1:1000, Cat No. 20146–1-AP), anti-Cyp1b1 (1:1000, Cat No. 18505–1-AP) or anti-β-actin (1:1000, Cat No. 20536–1-AP). After washing with TBST, the membrane was incubated with anti-rabbit IgG antibody (1:10,000, Cat No. SA00002-2) for 3 h. Finally, protein was analysed by chemiluminescence and quantitative analysis using ImageLab software (Bio-Rad). All antibodies were purchased from Proteintech (Proteintech Group, Inc, USA).

### Statistical analysis

All data were analysed via SPSS 17.0 (SPSS Inc., Chicago, USA) and expressed as the mean ± SD or median ± interquartile range (IQR) where appropriate. Data with normal distributions were analysed by one-way analysis of variance (ANOVA) or repeated measures analysis of variance, followed by an LSD post hoc test for determining the group differences. Data with nonnormal distributions were analysed using a nonparametric test (Kruskal–Wallis rank sum test), follow by Dunn’s post hoc test for determining the group differences. p < 0.05 was considered statistically significant.

## Results

### Effects of manual acupuncture (MA) on blood pressure (BP)

**Figure 1:**
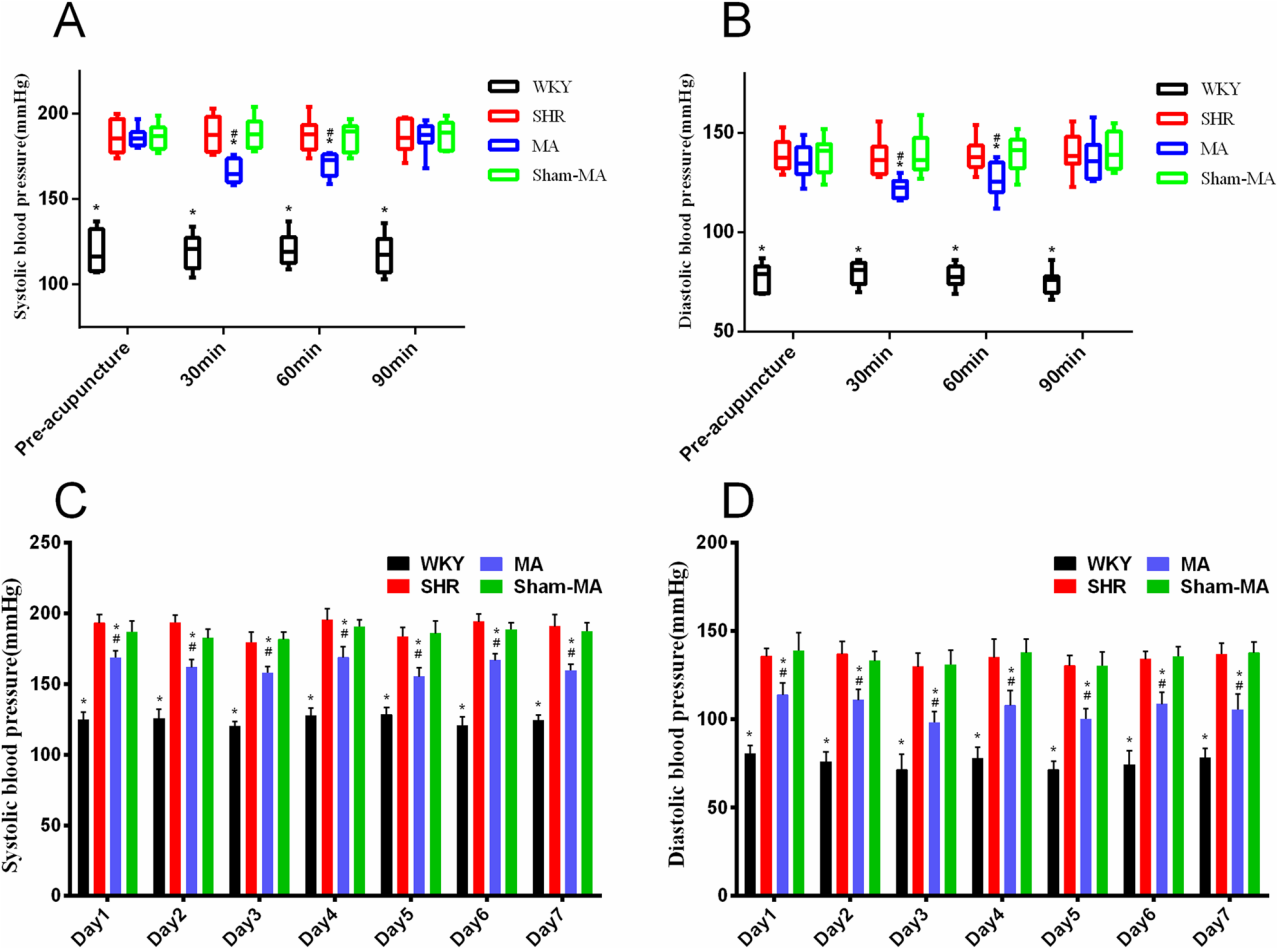
Reducing blood pressure (BP) effects induced by MA in SHRs. Differences in systolic blood pressure (SBP) (**A**) (H_(Pre-acupuncture)_ = 17.48, H_(30 min)_ = 25.68, H_(60 min)_ = 24.15, H_(90 min)_ = 17.48, Kruskal–Wallis Test) and (**C**) (F_(Day1)_ = 102.21, F_(Day2)_ = 101.11, F_(Day3)_ = 100.12, F_(Day4)_ = 103.36, F_(Day5)_ = 102.56, F_(Day6)_ = 108.47, F_(Day7)_ = 100.04, repeated measurement ANOVA) and diastolic blood pressure (DBP) (**B**) (H_(Pre-acupuncture)_ = 17.79, H_(30 min)_ = 25.32, H_(60 min)_ = 21.78, H_(90 min)_ = 18.12, Kruskal–Wallis Test) and (**D**) (F_(Day1)_ = 96.62, F_(Day2)_ = 97.40, F_(Day3)_ = 94.67, F_(Day4)_ = 95.72, F_(Day5)_ = 94.99, F_(Day6)_ = 98.54, F_(Day7)_ = 93.61, repeated measurement ANOVA) among WKY, SHR, MA and Sham-MA groups were detected at 30, 60 and 90 min after the first day of treatment, or every day of the 7-day treatment. *p < 0.05 versus the SHR group, ^#^p < 0.05 versus the Sham-MA group.

To evaluate the antihypertensive effect of MA, the BP of the rats was measured at 30, 60, and 90 min after the first treatment. As shown in Fig. 1A,B, the SBP and DBP of the MA group were significantly reduced at 30 and 60 min after acupuncture compared with those of the SHR group. Subsequently, for the following 6 days, the BP of the rats was repeatedly measured 30 min after daily acupuncture. Compared with the SHR group, acupuncture at KI3 significantly reduced the SBP and DBP of rats from the first day to the seventh day, as shown in Fig. 1C,D.

### Changes in glucose metabolism in the brain of SHRs

**Figure 2:**
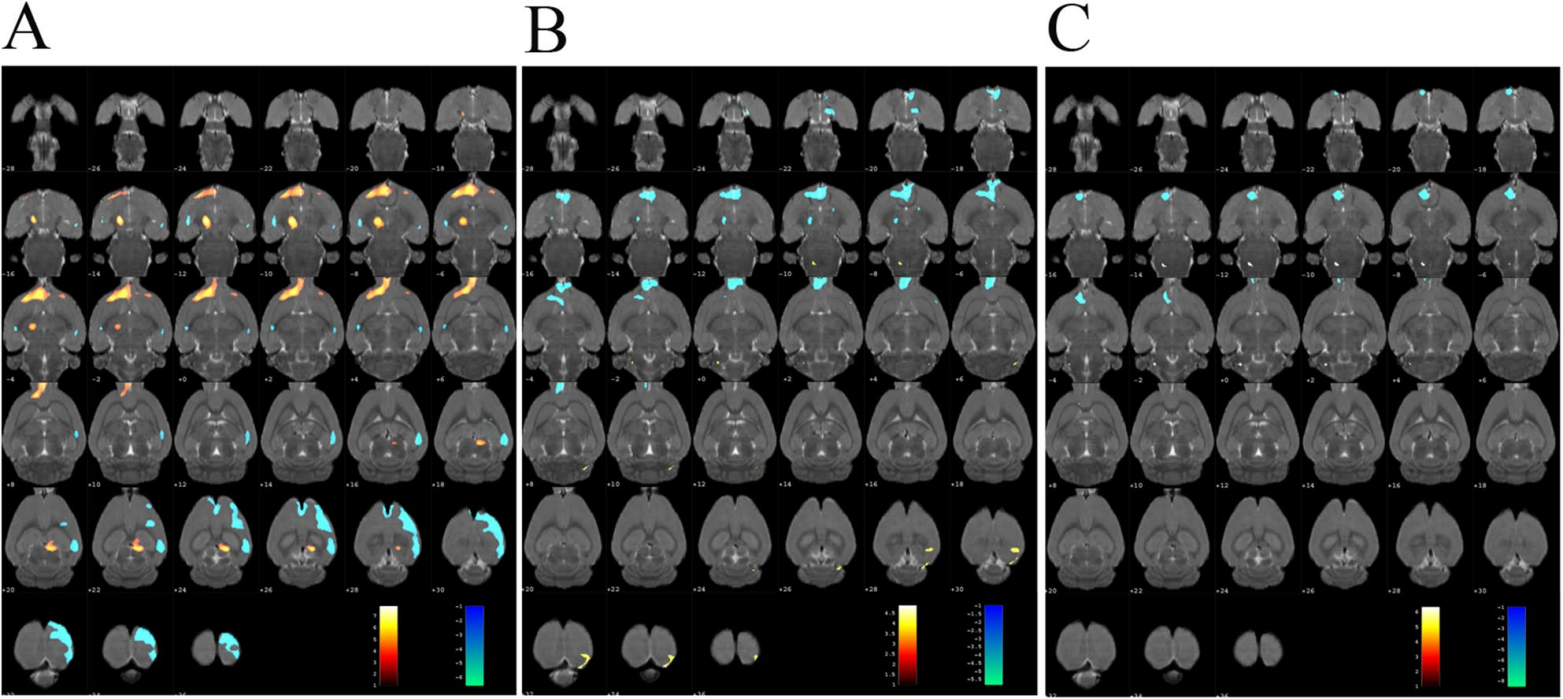
Changes of glucose metabolism in the rat brain. Regional glucose metabolism was scanned after the 7-day treatment. Results are overlaid on an axial view of the rat brain and mapped to the Paxinos and Watson rat brain atlas. (**A**) SHR group versus WKY group, (**B**) MA group versus SHR group, (**C**) Sham-MA group versus SHR group. Color bars represent the t-value of each significant voxel.

Glucose metabolism was significantly increased in the hypothalamus, thalamus, dorsal thalamus and olfactory bulb of the SHR group compared with the WKY group but decreased in other regions including the cingulate cortex, cingulate gyrus, and motor cortex (Table 2 and Fig. 2A). However, compared with the SHR group, the MA group showed significantly reduced glucose metabolism in the accumbens nucleus, hypothalamus, olfactory bulb, thalamus, anterior commissure, dorsal thalamus, and hypothalamus tuberal regions and increased glucose metabolism in the cerebellum posterior lobe and visual cortex (Table 3 and Fig. 2B). Furthermore, compared with the SHR group, the Sham-MA group showed significantly reduced glucose metabolism in the basal ganglia, caudate putamen, olfactory bulb, prefrontal cortex and orbital cortex, while glucose metabolism was increased in the medulla oblongata (Table 4 and Fig. 2C).

**Table 2:** Changes in brain glucose metabolism in the SHR group versus the WKY group.

Anatomical	Max_T	Peak coordinates(mm)		
		X	Y	Z
**Increased cerebral glucose metabolism**				
Agranular insular cortex	4.9318	− 3.5204	5.4957	2.7121
Accumbens nucleus	5.2113	− 1.4854	5.8600	3.4421
Hypothalamus	5.2084	5.2113	3.7700	− 5.1579
Infralimbic cortex	5.8891	− 1.7353	4.5220	3.6721
Lateral orbital cortex	6.1112	− 2.0434	4.2133	4.4821
Medial orbital cortex	4.4363	− 0.8938	3.6557	5.9021
Olfactory bulb	5.1011	− 0.9170	3.4705	7.7521
Thalamus	7.7016	− 2.1748	6.3023	− 3.2279
Ventral orbital cortex	6.5624	− 1.9962	3.8448	4.1621
Dorsal thalamus lateral nucleus group	7.7298	− 2.2315	6.2336	− 3.8979
Dorsal thalamus	7.7298	− 2.2315	6.2336	− 3.8979
Insular cortex	5.3416	− 3.8877	5.3742	2.4321
Nucleus around the septal area	5.3113	− 1.2554	5.8800	3.3821
Orbital cortex	6.2724	− 1.3262	4.5648	4.7821
Retrosplenial cortex	5.5247	0.9280	2.1260	− 5.9179
**Decreased cerebral glucose metabolism**				
Cingulate cortex	5.4028	− 0.4106	0.8324	2.8521
Cingulate gyrus	5.8715	− 0.8600	0.4552	2.4821
Motor cortex	6.7750	1.3706	− 0.1732	− 2.6879
Sensory cortex	6.4710	3.2213	0.4987	− 0.6379
Visual cortex	5.8175	5.7482	2.5629	− 5.5579

**Table 3:** Changes in brain glucose metabolism in the MA group versus the SHR group.

Anatomical	Max_T	Peak coordinates(mm)		
		X	Y	Z
**Increased cerebral glucose metabolism**				
Cerebellum posterior lobe	3.9995	3.6906	5.0247	− 10.8638
Visual cortex	5.0153	2.8525	0.9995	− 9.0979
**Decreased cerebral glucose metabolism**				
Accumbens nucleus	5.0146	− 2.1170	5.5260	2.3221
Hypothalamus	4.0081	0.7951	7.9986	− 1.9379
Olfactory bulb	6.1332	− 1.0071	3.6408	6.1621
Prefrontal cortex	5.5397	− 1.1074	4.0621	5.9221
Thalamus	4.5132	− 2.1388	5.8601	− 3.0379
Anterior commissure	5.1151	− 0.8343	4.2171	6.1621
Dorsal thalamus	4.1132	− 1.9388	6.5601	− 2.9379
Hypothalamus tuberal region	3.8469	0.9891	8.3124	− 2.3779
Orbital cortex	5.0835	− 0.8844	3.3049	5.8421
Striatum	4.2395	− 2.7483	6.1624	1.9821

**Table 4:** Changes in brain glucose metabolism in the Sham-MA group versus the SHR group.

Anatomical	Max_T	Peak coordinates(mm)		
		X	Y	Z
**Increased cerebral glucose metabolism**				
Medulla oblongata	6.6063	− 1.6222	7.9011	− 11.9179
**Decreased cerebral glucose metabolism**				
Anterior olfactory nucleus	6.5951	− 1.1251	6.0005	3.2421
Accumbens nucleus	8.8591	− 1.4525	5.7490	2.2221
Basal ganglia	6.4873	− 1.6531	5.2821	2.7621
Caudate putamen	6.4873	− 1.6531	5.2821	2.7621
Olfactory bulb	6.0483	− 1.4155	3.7857	6.8621
Prefrontal cortex	7.0097	− 1.1187	5.8147	2.9421
Anterior commissure	6.9999	− 1.7768	6.5065	2.0421
Nucleus around the septal area	8.3091	− 1.5325	5.8849	2.4421
Orbital cortex	5.9403	− 1.5326	4.7666	3.7221

### Filtering of raw sequencing reads

**Figure 3:**
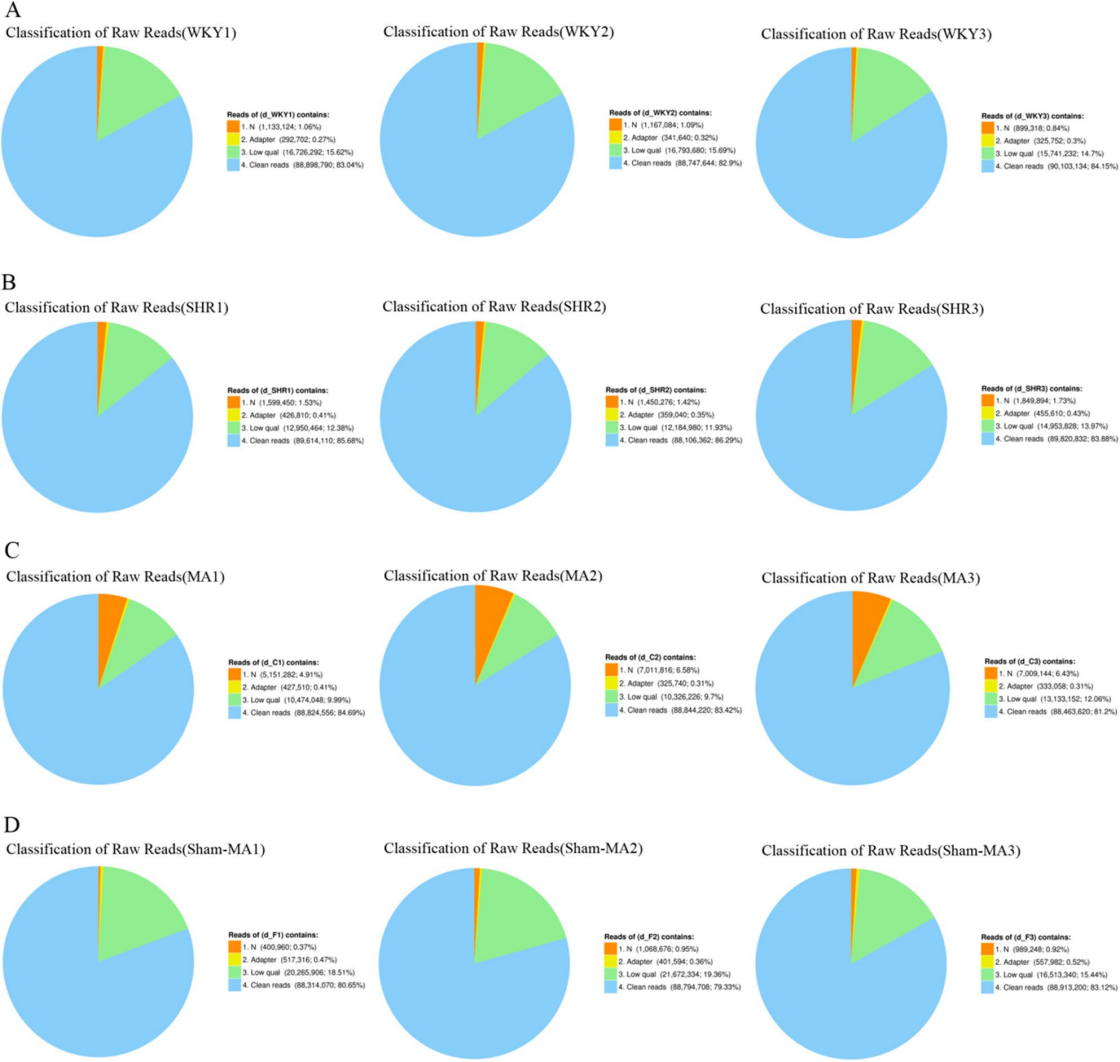
A summary of the original RNA-Seq data. The original data performance of the WKY (**A**), SHR (**B**), MA (**C**) and Sham-MA (**D**) group is shown in the pie chart (n = 3). N: The total amount of reads that contain more than 5% unknown N base (the N reads ratio); adaptor: The total amount of reads that contain adaptors (the adaptor ratio); low quality: More than 20% of the bases in the total read have a quality score lower than 15 (a low quality read ratio); clean reads: Reads filtered with N reads; reads have adaptors and low quality reads (a clean read ratio).

Transcriptomic sequencing was conducted to analyse the expression profile of the hypothalamus in the WKY, SHR, and MA groups. A total of 955.27 Mbp of raw reads were obtained. The percentage of clean reads Q20 and Q30 (the ratio of the quality values of the reads that were respectively larger than 20 and 30, respectively, compared to the total reads) indicated that the sequence was of high quality and could be used for subsequent analyses (Table 5 and Fig. 3). After filtering the invalid readings, 267.75, 267.54, 266.12 and 266.01 Mbp of clean readings were obtained from the WKY, SHR, MA and Sham-MA groups, respectively. The proportion of clean reads was 82.9–84.15% in the WKY group, 84.15–86.29% in the SHR group, 81.2–84.69% in the MA group and 79.33–83.12% in the Sham-MA group. The clean read ratio of the rat genome suggested that the sequencing depth was satisfactory for the analysis of differentially expressed genes between the groups of rats.

**Table 5:** Quality metrics f clean reads.

Sample	Total raw	Total clean	Total clean	Clean reads	Clean reads	Clean reads
	Reads (Mbp)	Reads (Mbp)	Bases (Gbp)	Q20 (%)	Q30 (%)	Ratio (%)
WKY1	107.05	88.9	8.89	97.64	91.29	83.04
WKY2	107.05	88.75	8.87	97.56	91.05	82.9
WKY3	107.07	90.1	9.01	97.74	91.44	84.15
SHR1	104.59	89.61	8.96	97.7	91.34	85.68
SHR2	102.1	88.11	8.81	97.76	91.47	86.29
SHR3	107.08	89.82	8.98	97.42	90.86	83.88
MA1	104.88	88.82	8.88	97.66	91.52	84.69
MA2	106.51	88.84	8.88	97.63	91.39	83.42
MA3	108.94	88.46	8.85	97.35	90.6	81.2
Sham-MA1	109.5	88.31	8.83	96.44	89.3	80.65
Sham-MA2	111.94	88.79	8.88	96.45	89.42	79.33
Sham-MA3	106.97	88.91	8.89	96.83	90.34	83.12

### Differentially expressed genes in the hypothalamus of WKY, SHR, MA and Sham-MA rats

**Figure 4:**
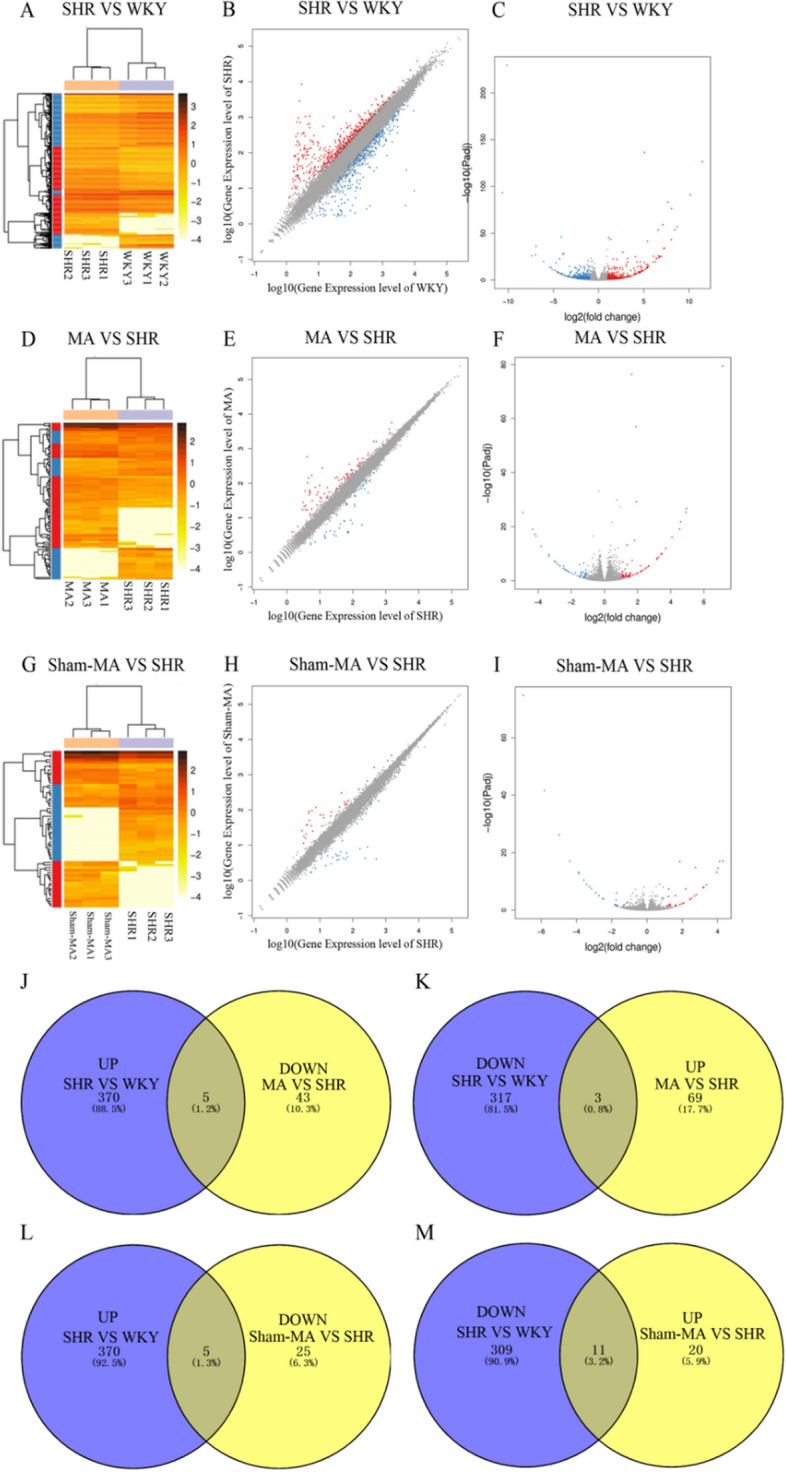
Significantly different mRNA expression in the PVH of WKY, SHR, MA and Sham-MA. The hierarchical clustering (**A**,**D**,**G**), scatter plot (**B**,**E**,**H**), and volcano plot (**C**,**F**,**I**) show differentially expressed genes (DEGs) between the WKY, SHR, MA and Sham-MA groups with the red and blue colors, respectively suggesting upregulated or downregulated expression; (**J**–**M**) Venn diagrams of the overlapping DEGs between groups are shown. Five genes increased in the SHR group but decreased in the MA group. Three genes decreased in the SHR group but increased in the MA group. The analysis of Volcano-plots, Scatter-plots and heatmaps were performed using R version 3.6.0 (https://cran.r-project.org/bin/windows/base/old/3.6.0/). The overlap in upregulated and downregulated gene expression between the different groups was analysed using Venn online software VENNY version 2.1 (https://bioinfogp.cnb.csic.es/tools/venny/index.html).

To study the regulation of DEGs between the WKY, SHR, MA and Sham-MA groups, DEGs in the hypothalamus were analysed. DESeq2 algorithms were based on a negative binomial distribution to detect the DEGs by applying a fold change ≥ 2.00 and an adjusted p-value (p ≤ 0.05). The statistics of the number of DEGs are shown by a heatmap (Fig. 4A,D,G), scatter plot (Fig. 4B,E,H), and volcano plot (Fig. 4C,F,I). There were 695 DEGs in the SHRs relative to the WKY rats, with 375 upregulated genes and 320 downregulated genes. A total of 120 DEGs were found in the MA rats relative to the SHRs, with 72 upregulated genes and 48 downregulated genes. A Venn analysis was used to study the possible genes involved in reducing blood pressure as a result of acupuncture. We found that MA treatment abolished the upregulation of 5 of the 375 genes upregulated in SHRs compared to wild-type rats. Correspondingly, MA treatment was able to counteract the downregulation of 3 of the 320 downregulated genes in SHRs (Fig. 4J,K). However, DEGs that were up- or downregulated by sham acupuncture were not involved in the blood pressure regulation process(Fig. 4L,M).

### Gene ontology and Kyoto encyclopedia of genes and genomes analysis

**Figure 5:**
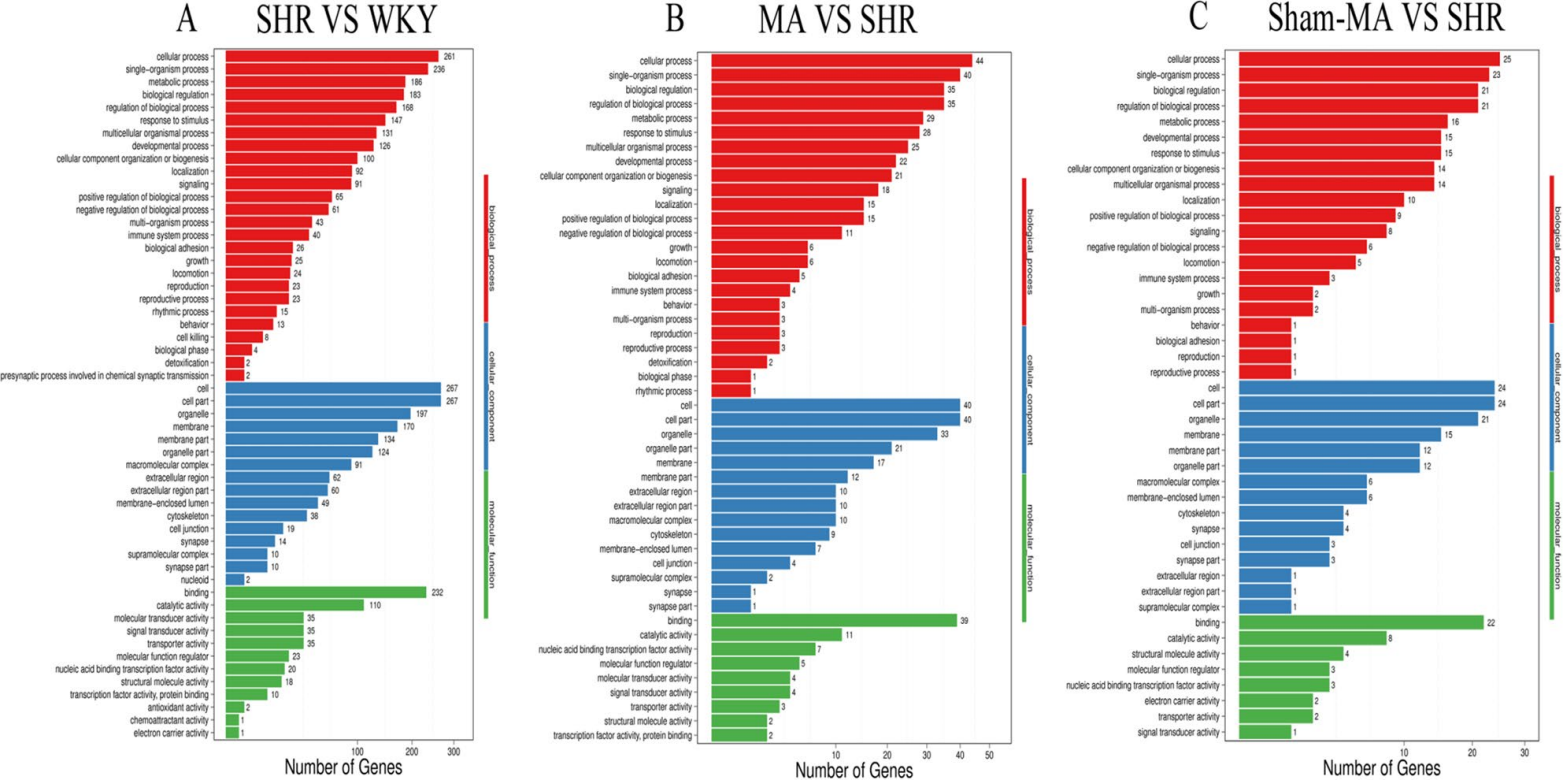
The DEGs number of the most enriched Gene Ontology (GO) terms between WKY, SHR, MA and Sham-MA. (**A**) SHR versus WKY; (**B**) MA versus SHR; (**C**) Sham-MA versus SHR. GO analyses were performed using enrichment analysis with the phyper function in R version 3.6.0 (https://cran.r-project.org/bin/windows/base/old/3.6.0/).

To investigate the function of the DEGs, Gene Ontology (GO) classification and functional enrichment were performed. GO covers three domains: biological process, cellular component, and molecular function. Functional enrichment was performed and the GO classification results between the WKY, SHR, MA and Sham-MA groups are shown in Fig. 5A–C. Regarding biological processes, the categories “cellular process”, “single-organism process”, “metabolic process”, and “biological regulation” showed a high degree of enrichment. The DEGs were involved in the “cell”, “cell part”, “organelle”, and “membrane” categories according to their cellular component classification. In terms of the molecular functions, “molecular transducer activity”, “binding”, “catalytic activity”, and “nucleic acid-binding transcription factor activity” showed a high degree of enrichment.

**Figure 6:**
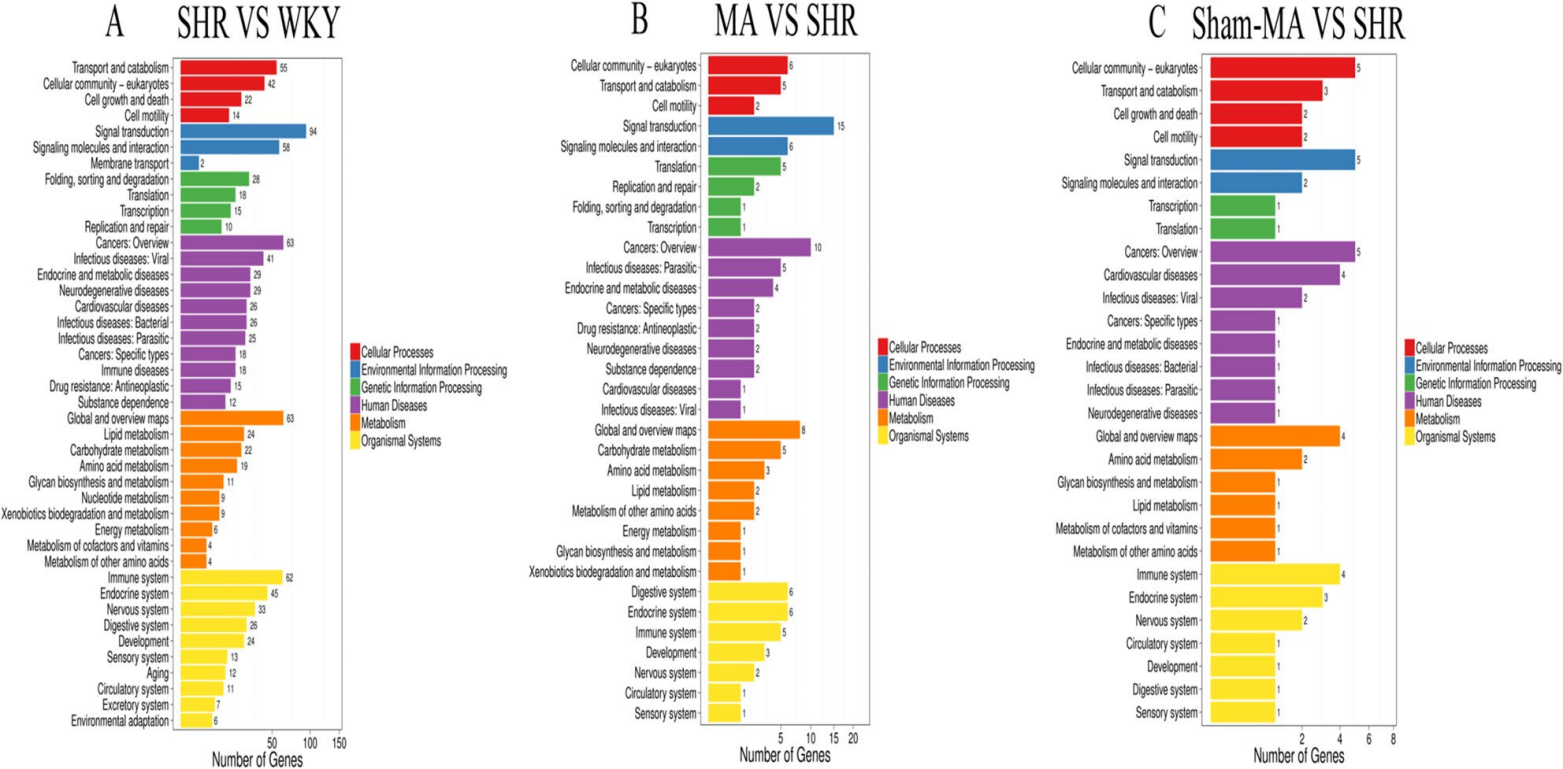
The KEGG Pathway functional enrichment results for the DEGs between WKY, SHR, MA and Sham-MA. With DEGs, KEGG pathway classification and functional enrichment were performed. (**A**) SHR versus WKY; (**B**) MA versus SHR; (**C**) Sham-MA versus SHR. KEGG analyses were performed using enrichment analysis with the phyper function in R version 3.6.0 (https://cran.r-project.org/bin/windows/base/old/3.6.0/).

To further investigate the possible pathways affected directly by MA treatment in SHRs, DEGs were classified by performing KEGG pathway classification and functional enrichment. The terms with false discovery rates (FDRs) no larger than 0.01 are defined as being significantly enriched. As shown in Fig. 6, DEGs were found to be enriched in several signalling pathways, including “endocrine and metabolic diseases”, “neurodegenerative diseases”, “cardiovascular diseases”, “energy metabolism”, and “signaling molecules and interaction”.

### Validation of the differentially expressed genes using real-time PCR and Western blot

**Figure 7:**
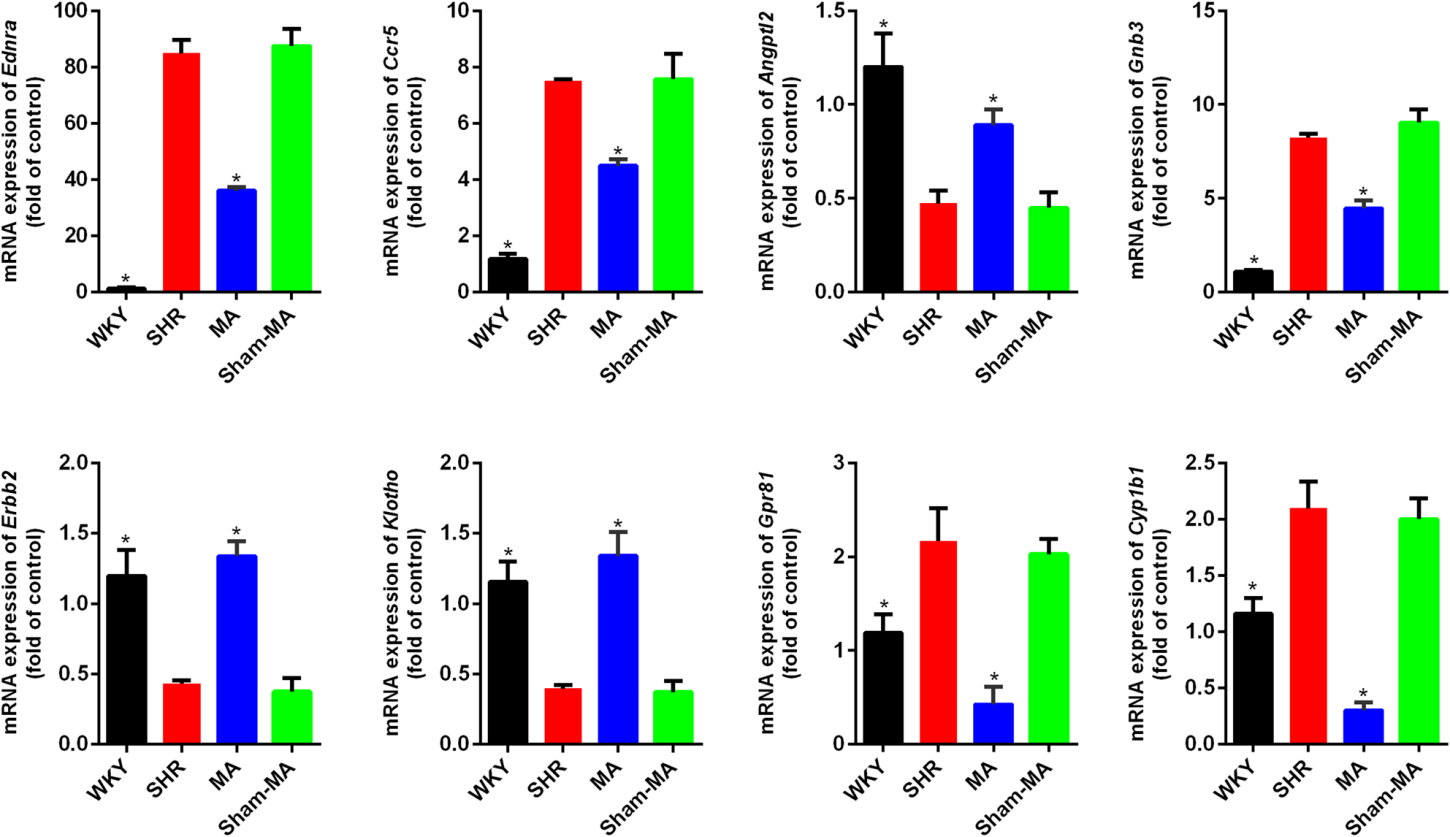
Validation of the up/downregulated genes using qRT-PCR. The loading control gene β-actin was used for normalization. Data are expressed as the mean ± SD. *p < 0.05 versus the SHR group (F_(Ednra)_ = 30.94, F_(Ccr5)_ = 119.78, F_(Angptl2)_ = 74.93, F_(Gnb3)_ = 336.43, F_(Erbb2)_ = 53.47, F_(Klotho)_ = 215.43, F_(Gpr81)_ = 35.09, F_(Cyp1b1)_ = 54.32, one way-ANOVA).

To verify the reliability of the RNA-Seq data, real-time PCR was used to examine the expression of eight DEGs: *Angptl2, Erbb2, Klotho, Ednra, Ccr5, Gnb3, Gpr81*, and *Cyp1b1*. As shown in Fig. 7, trends in the expression data of the examined DEGs were the same as those observed for RNA-Seq. Three genes, *Angptl2*, *Erbb2*, and *Klotho*, were downregulated in the SHR group but upregulated after MA treatment. On the other hand, five genes, *Ednra, Ccr5, Gnb3, Gpr81* and *Cyp1b1* were upregulated in the SHR group but downregulated after MA treatment. However, Sham-MA did not significantly regulate these DEGs.

**Figure 8:**
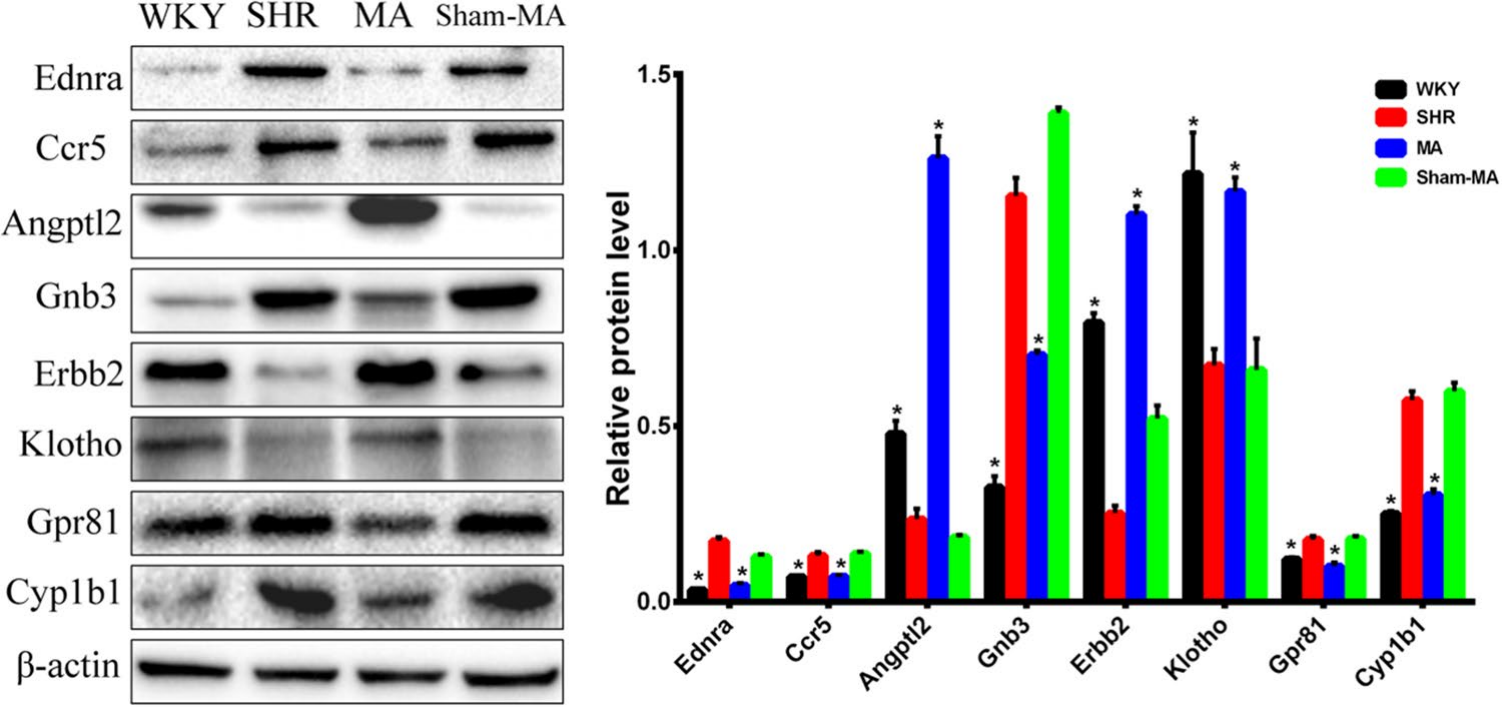
Validation of the up/downregulated genes using western blot. The loading control protein β-actin was used for normalization. Data are expressed as the mean ± SD. *p < 0.05 versus the SHR group (F_(Ednra)_ = 483.27, F_(Ccr5)_ = 82.53, F_(Angptl2)_ = 254.11, F_(Gnb3)_ = 236.63, F_(Erbb2)_ = 507.16, F_(Klotho)_ = 655.65, F_(Gpr81)_ = 60.18, F_(Cyp1b1)_ = 43.76, one way-ANOVA).

To further confirm that the regulated DEGs lead to protein modulation and explain the potential mechanisms underlying the acupuncture effects, western blotting was used to examine the protein levels of the DEGs (Fig. 8). Consistent with the real-time RT-PCR results and as expected, the results of western blotting showed that the protein levels of Ednra, Ccr5, Gnb3, Gpr81 and Cyp1b1 were downregulated in MA-treated rats compared to SHRs, the protein levels of Angptl2, Erbb2 and Klotho were upregulated in the MA group. Interestingly, the protein level of eight DEGs was not regulated after Sham-MA treatment.

## Discussion

Essential hypertension is the most common form of hypertension^23^. Although laboratories in many countries have studied the basic mechanism of acupuncture, little is known about its mechanism of regulating blood pressure. Our research results show that within 30 to 60 min after acupuncture treatment, SBP and DBP of SHRs are effectively reduced. Clinical acupuncture usually involves repeated acupuncture treatments. This study also found that the antihypertensive effect of acupuncture can be stably repeated within a 7-day treatment cycle. In fact, repeated acupuncture may produce more molecular changes and longer-term cardiovascular effects than a single bout of acupuncture^24^. Our research shows that acupuncture is a potential method in the treatment of hypertension, and its mechanism is worthy of in-depth study.

To reveal the central mechanism related to the control of blood pressure by acupuncture, we used PET-CT to detect changes in brain glucose metabolism in SHRs. Interestingly, compared with that in the WKY group, the glucose metabolism of PVH in the SHR group was upregulated, and the upregulation was reversed by acupuncture. However, sham acupuncture failed to play a similar regulatory role. Therefore, we speculate that only a few specific acupoints can regulate blood pressure, which is consistent with existing research results^24^. We know that PVH can regulate many human diseases, such as hypertension, obesity and diabetes^25–27^. In addition, increased sympathetic outflow from the PVH and excessive activation of sympathetic synapse N-methyl-D-aspartate receptors are critical to hypertension^28^. Our PET-CT results suggest that PVH may be one of the target brain regions for acupuncture KI3 to regulate blood pressure. To further explore the influence of acupuncture on the molecular mechanism affecting the PVH, we then performed PVH transcriptome sequencing.

After statistical analysis of the PVH transcriptome sequencing data, we found that compared with the WKY group, the expression of the Angptl2, Erbb2 and Klotho genes in the PVH of the SHR group decreased, while the expression of Ednra, Ccr5, Gnb3, Gpr81 and Cyp1b1 increased, and acupuncture at KI3 reversed the expression changes of these DEGs. According to the GO functional classification, the DEGs were enriched in the categories of “catalytic activity”, “transporter activity”, “molecular function regular”, and “signal transducer activity”. KEGG analysis revealed that the DEGs were involved in several pathways, including the “endocrine system”, “cardiovascular diseases”, and “neurodegenerative diseases”.

However, the exact mechanisms of the involvement of these DEGs in the regulation of blood pressure by acupuncture at KI3 remain unclear. Therefore, we combined the DEGs in the PVH verified by qPCR and western blot in this study with the reported mechanisms of acupuncture-induced lowering of blood pressure to preliminarily clarify the potential central blood pressure regulatory mechanism of acupuncture at KI3.

The DEG Klotho was upregulated in the PVH after MA at KI3. As a newly discovered antiaging gene, Klotho is mainly expressed in the brain and kidney^29,30^. In the kidney, the mechanism by which the Klotho protein regulates blood pressure has been extensively researched. For example, Klotho may exert beneficial antihypertensive activity by inhibiting the activation of the renin-angiotensin system (RAS) and the production of systemic aldosterone. Klotho can inhibit intrarenal RAS by targeting Wnt/β-catenin signalling to prevent Wnt-induced nuclear translocation of β-catenin to lower blood pressure^31^. Researchers found that phosphate may be an independent cause of hypertension, and Klotho is selectively expressed in the distal and proximal tubules to control the reabsorption of phosphate^32^. Recently, researchers have noted the relationship between Klotho and blood pressure regulation in the CNS. The latest research has shown that Klotho can inhibit the level of oxidative stress^33^. Hypertension is related to high levels of oxidative stress in the PVH. Oxidative stress is mainly manifested as an increase in the formation of reactive oxygen species (ROS). This abnormal increase precedes the formation of hypertension in SHRs and is related to the occurrence and maintenance of hypertension^34^. Our previous studies revealed that acupuncture could regulate the expression of 7 proteins related to oxidative stress in the medulla oblongata of SHRs^35^, but the exact regulatory mechanism was still unclear. Based on our results, we speculated that acupuncture at KI3 might upregulate Klotho to inhibit hypothalamic oxidative stress levels, thereby downregulating the blood pressure of SHRs. In addition, Klotho encodes transmembrane glycoproteins with extracellular domains, including Klotho, αKlotho and βKlotho proteins, which are important parts of the endocrine fibroblast growth factor 21 (FGF21) receptor complex^36^. FGF21 has a wide range of biological functions^37^. It can cross the blood–brain barrier and bind to the Klotho protein in the PVH to form a complex. This complex activates the HPA axis and regulates the release of corticosterone by acting on hypothalamic neurons. It is worth noting that corticosterone has been widely reported to be related to the suppression of hypertension. According to our data, we speculated that upregulating Klotho in the PVH can promote the formation of the FGF21-Klotho complex, activate the HPA axis to release corticosterone, and ultimately lower blood pressure, which may be a potential central mechanism by which acupuncture at KI3 reduces blood pressure.

Our study showed that acupuncture at KI3 can upregulate the expression of Erbb2 in the PVH of SHRs. The Erbb2 protein is a subtype of the tyrosine kinase receptor family, and it participates in NRG-1/Erbb signalling along with neuromodulin 1 (NRG-1) located in the central nervous system(CNS). Studies have revealed that NRG-1/Erbb signals in the CNS play an important role in cardiovascular homeostasis^38^, and activating the NRG-1/Erbb signalling in the CNS can reduce blood pressure, heart rate and renal sympathetic nerve activity^38^. The expression level of Erbb2 in the brainstem of SHRs was significantly lower than that in WKY rats. Inhibiting the expression of Erbb2 could increase blood pressure and heart rate^39^. Studies have shown that reducing the level of Erbb2 in the rat brain causes hypertension by reducing NO synthesis and inhibiting the activity of γ-aminobutyric acid^40^. According to existing research, Erbb receptors and NRG-1 are widely distributed in the central and peripheral nervous systems^41,42^. Our research showed that, compared with that of WKY rats, the expression of Erbb2 in the PVH of SHRs decreased, while acupuncture at KI3 can upregulated the expression of Erbb2 in the PVH. We speculated that acupuncture at KI3 could upregulate Erbb2 in the PVH, activate NRG-1/Erbb signalling and inhibit sympathetic nerve activity, thereby decresing blood pressure and heart rate. This may be one of the central mechanisms by which acupuncture regulates blood pressure in SHRs.

CCR5 is a chemokine receptor. Our results showed that CCR5 was highly expressed in the PVH in SHRs, while acupuncture at KI3 could downregulated its expression. Studies have shown that CCR5 can mediate the activation of the NF-κB pathway, while the inflammation mediated by the NF-κB pathway can be inhibited by knocking out or inhibiting the expression of CCR5^43^. Inflammation in the PVH can induce an increase in blood pressure. Under pathological conditions, the PVH drives inflammation through activation of NF-κB, thereby increasing the activity of the renin-angiotensin system, leading to hypertension. In addition, activation of NF-κB can also increase the release of the downstream inflammatory cytokine TNF-α, which in turn increases sympathetic nerve outflow to raise blood pressure. Abnormally elevated TNF-α may also increase the permeability of the blood–brain barrier, allowing more angiotensin to enter the brain, and at the same time the loss of reflex baroreceptor function leading to increased blood pressure^44^. Our results showed that CCR5 in the PVH was inhibited after acupuncture at KI3, which suggested that acupuncture may reduce blood pressure by inhibiting the NF-κB pathway and inflammation of the hypothalamus. On the other hand, CCR5 can also regulate the MAPK pathway. Pharmacological experiments have shown that downregulating CCR5 mediates the degradation of MAPK, IKK and IκBα, and enhances the inhibitory effect of the inflammatory response^45^. Inhibition of MAPK signal transduction in the PVH has been shown to prevent sympathetic nerve overexcitation^46^. Therefore, downregulating the expression of CCR5 in the PVH can improve sympathetic hyperexcitability by inhibiting MAPK-mediated inflammatory pathways, thereby reducing blood pressure. In summary, we believe that the increased expression of CCR5 in the PVH may be one of the mechanisms of the SHR blood pressure increase, and that CCR5 may mediate the activation of the NF-κB and MAPK signalling pathways and the renin-angiotensin system to increase the sympathetic nerve outflow, eventually leading to high blood pressure. In this study, the qPCR and western blot results showed that acupuncture at KI3 downregulated the expression of CCR5, so we speculated that acupuncture may reduce CCR5-mediated inflammation of the PVH and reduce sympathetic nerve outflow to regulate blood pressure.

In addition to the expression of the above genes, that of Angptl2, CYP1B1, GPR81, EDNRA, and GNB3 can also be regulated by acupuncture at KI3. Angiopoietin-like protein 2 (Angptl2) belongs to the angiopoietin-like family. Studies have shown a correlation between the level of circulating Angptl2 and the diagnosis and/or prognosis of cardiovascular disease^47^. Cytochrome P-4501B1 (CYP1B1) induces reactive oxygen species (ROS) to cause inflammation, cardiovascular hypertrophy and hypertension-related endothelial dysfunction, which is mediated by the activation of ERK1/2 and c-SRC signalling pathways^48^. GPR81 belongs to the family of hydroxycarboxylic acid receptors, and existing studies have provided evidence for the potential new role of GPR81 agonists in blood pressure control and renal vascular resistance regulation (including the regulation of the endothelin system, a known vascular effect mechanism). However, the expression level of GPR81 in the brain is low, and it seems to be involved in neuronal signalling^49^. Endothelin receptor type A (EDNRA) combined with vasoconstrictors released by endothelial cells can increase vasoconstriction and sodium retention, leading to increased blood pressure. The use of EDNRA selective antagonists can reduce blood pressure^50^. The G protein β3 (GNB3) subunit is involved in G protein coupled receptor signal transduction. The GNB3 C825T polymorphism is a marker for the treatment of hypertension, and its phenotype has enhanced sodium-proton reverse motility activity. The increase in essential hypertension is related to the T allele of the GNB3 C825T polymorphism^51^. However, to date, no studies have found roles of these genes in central tissues, such as the PVH. Therefore, roles of these DEGs in the PVH and their signalling pathways participating in lowering blood pressure caused by acupuncture at KI3 need to be confirmed in further studies.

One limitation in this study was that the blood pressure measurements were tail-cuff blood pressure measurements instead of radiotelemetry blood pressure measurements. When using this type of measurement, rats may be in a state of stress, which causes the blood pressure measurement value to deviate from the true value slightly.

## Conclusion

Our data suggest that acupuncture at KI3 can effectively reduce the blood pressure of spontaneously hypertensive rats. PET-CT scanning results further indicated that the PVH could be one of the target brain areas for MA at KI3 to manage blood pressure. Furthermore, whole transcriptome sequencing of the PVH was used to explore how DEGs regulated by acupuncture in SHRs are related to cardiovascular diseases, thereby providing a theoretical basis for the further study of acupuncture treatment of hypertension (Supplementary Information).

## Supplementary Information

Supplementary Information.
