# Supplementary material for: PET-CT and RNA sequencing reveal novel targets for acupuncture-induced lowering of blood pressure in spontaneously hypertensive rats
Source: Sci Rep. 2021 May 26;11:10973. doi: 10.1038/s41598-021-90467-1 (PMC8155206; doi:10.1038/s41598-021-90467-1)

PET-CT and RNA sequencing reveal novel targets for acupuncture-induced lowering of blood pressure in spontaneously hypertensive rats

Jing Li[1, 2, 4], Chong Peng[2, 5], Dongjian Lai[4], Yajing Fang[3], Daihong Luo[6], Zunming Zhou[5], Chenyun Li[4], Xinsheng Lai[3, 4*]

1. Integrative Cancer Centre, The First Affiliated Hospital of Guangzhou University of Chinese Medicine, Guangzhou, Guangdong, 510405, China

2. Postdoctoral Research Station of Guangzhou University of Chinese Medicine, Guangzhou, Guangdong, 510405, China

3. Rehabilitation Center, The First Affiliated Hospital of Guangzhou University of Chinese Medicine, Guangzhou, Guangdong, 510405, China

4. Clinical School of Acupuncture and Rehabilitation, Guangzhou University of Chinese Medicine, Guangzhou, Guangdong, 510405, China

5. Department of Gastroenterology, The First Affiliated Hospital of Guangzhou University of Chinese Medicine, Guangzhou, Guangdong, 510405, China

6. Shenzhen Hospital of Beijing University of Chinese Medicine, Shenzhen, Guangdong, 518100, China

*Correspondence: lai1023@163.com

Original and unprocessed versions of western blot in Fig.8

1.Ednra

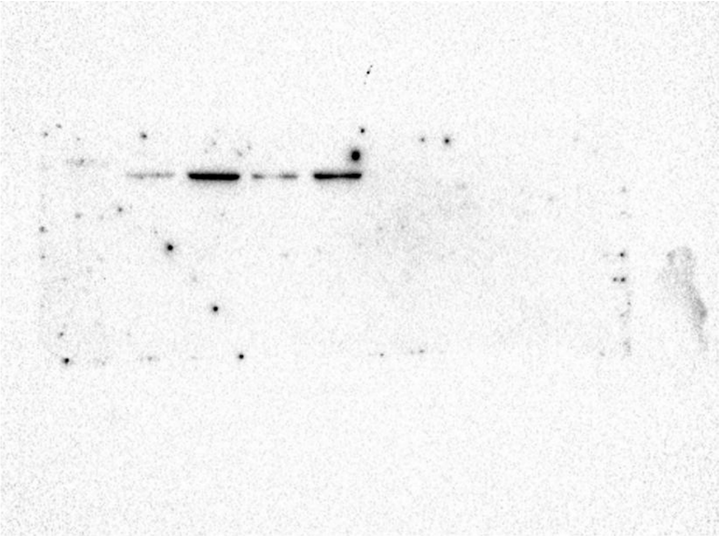

2. Ccr5

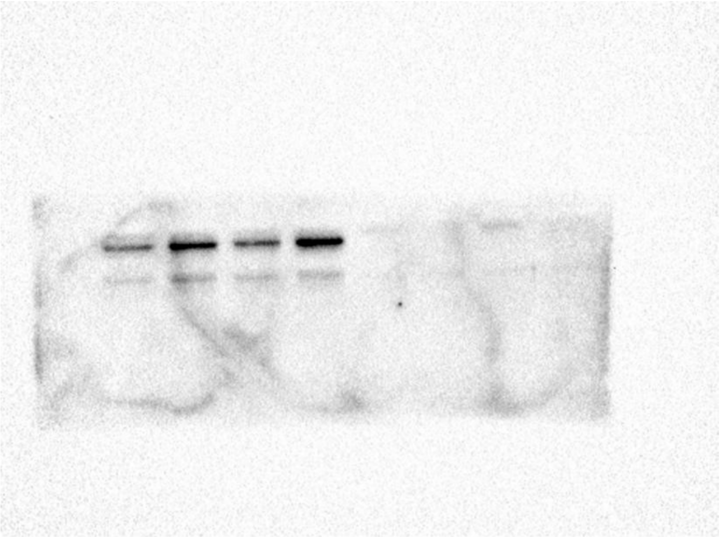

3. Angptl2

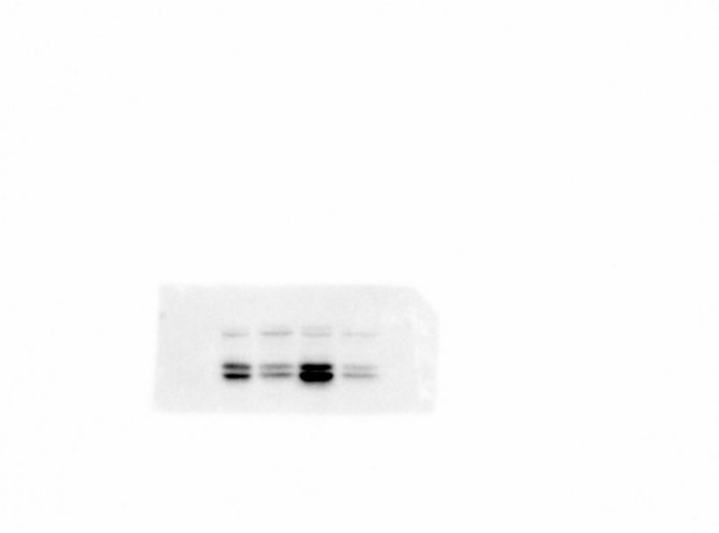

4. Gnb3

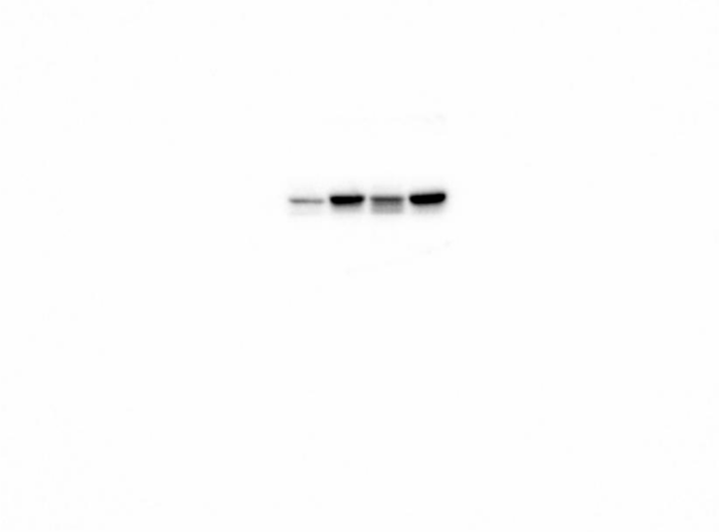

5. Erbb2

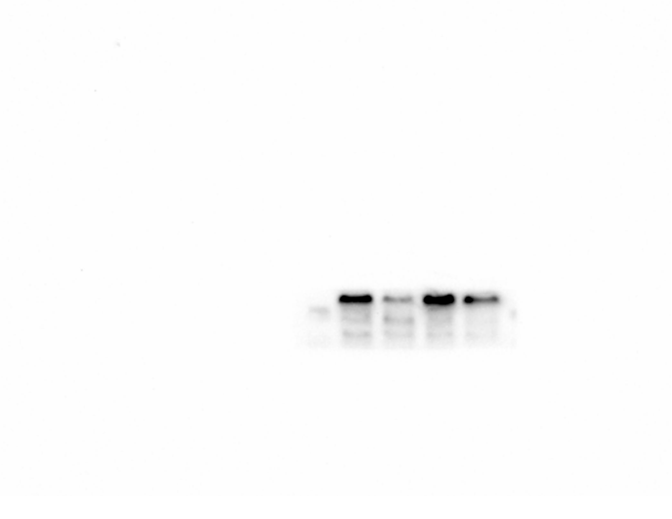

6. Klotho

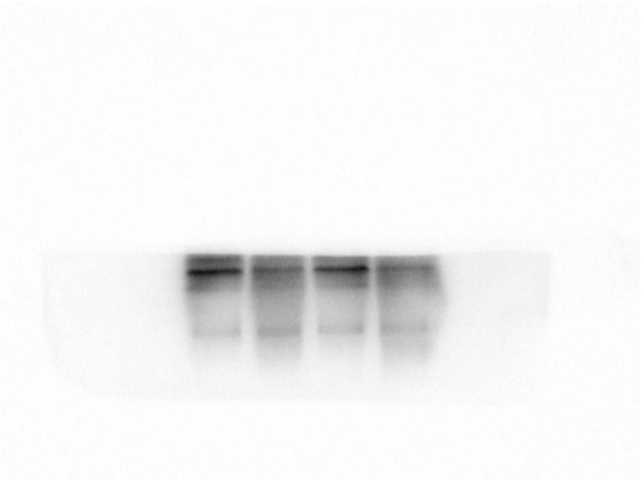

7.Gpr81

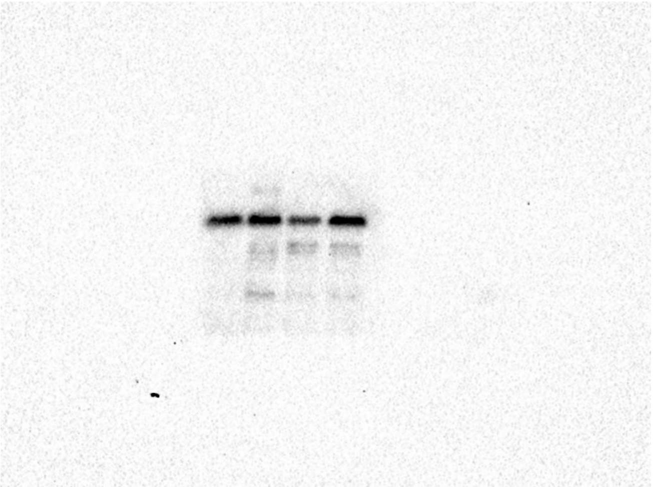

8.Cyp1b1

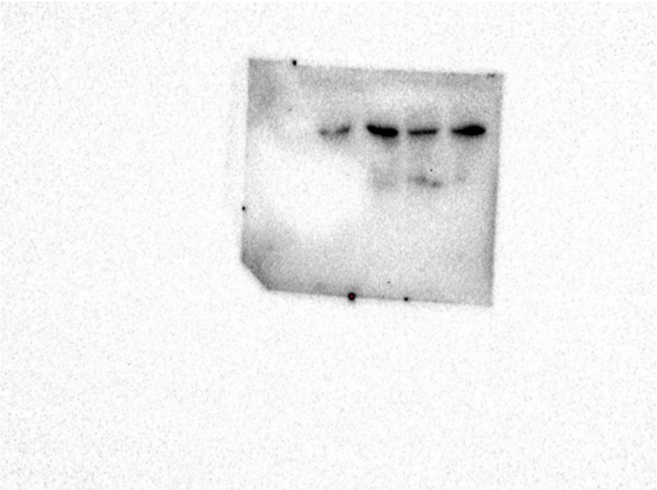

9.β-actin

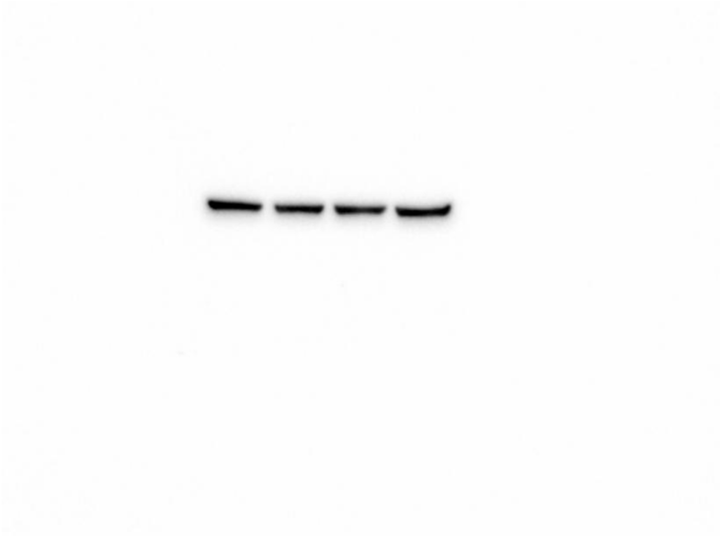

Supplement: Supplementary file 1 — Supplementary Information. [file 41598_2021_90467_MOESM1_ESM.pdf]
